# Fecal incontinence after total mesorectal excision for rectal cancer—impact of potential risk factors and pelvic intraoperative neuromonitoring

**DOI:** 10.1186/s12957-020-1782-6

**Published:** 2020-01-15

**Authors:** Daniel W. Kauff, Yvonne D. S. Roth, Rika S. Bettzieche, Werner Kneist

**Affiliations:** 1 Department of General, Visceral and Transplant Surgery, University Medicine of the Johannes Gutenberg University Mainz, Mainz, Germany; 2 Department of General and Visceral Surgery, St. Georg Hospital Eisenach gGmbH, Mühlhäuser Straße 94, 99817 Eisenach, Germany

**Keywords:** Rectal cancer, Autonomic nervous system, Neoadjuvant therapy, Fecal incontinence, Intraoperative monitoring

## Abstract

**Background:**

Fecal incontinence frequently occurs after total mesorectal excision for rectal cancer. This prospective study analyzed predictive factors and the impact of pelvic intraoperative neuromonitoring at different follow-up intervals.

**Methods:**

Fifty-two patients were included undergoing total mesorectal excision for rectal cancer, and 29 under control of pelvic intraoperative neuromonitoring. Fecal incontinence was assessed using the Wexner Score at 3 and 6 months after stoma closure (follow-ups 1 and 2) as well as 1 and 2 years after surgery (follow-ups 3 and 4). Risk factors were identified by means of logistic regression.

**Results:**

New onset of fecal incontinence was significantly lower in the neuromonitoring group at each follow-up (follow-up 1: 2 of 29 patients (7%) vs. 8 of 23 (35%), (*p* = 0.014); follow-up 2: 3 of 29 (10%) vs. 9 of 23 (39%), (*p* = 0.017); follow-up 3: 5 of 29 (17%) vs. 11 of 23 (48%), *p* = 0.019; follow-up 4: 6 of 28 (21%) vs. 11 of 22 (50%), *p* = 0.035).

Non-performance of neuromonitoring was found to be an independent predictor for fecal incontinence throughout the survey. Neoadjuvant chemoradiotherapy was an independent predictor in the further course 1 and 2 years after surgery.

**Conclusions:**

Performance of pelvic intraoperative neuromonitoring is associated with significantly lower rates of fecal incontinence. Neoadjuvant chemoradiotherapy was found to have negative late effects. This became evident 1 year after surgery.

## Background

Bowel dysfunction after total mesorectal excision (TME) for rectal cancer occurs frequently. It seriously impacts patients’ daily lives leading not only to physical but also to emotional suffering with persistent feelings of insecurity. The dysfunction rate ranges from 50 to 90%. Anorectal dysfunction includes fecal incontinence (FI), urgency, frequency, and stool clustering, a symptom complex summarized under “low anterior resection syndrome” (LARS) [1–3].

Several instruments were used for evaluation of postoperative bowel dysfunction. This resulted in substantial variations in the reporting of the abovementioned symptoms. The most frequently used instrument is the validated Wexner Score focusing on FI [4].

A variety of risk factors for FI have been reported such as low rectal cancer, radiotherapy, and pelvic autonomic nerve damage [5–7]. In particular, the combination of a low-lying tumor and neoadjuvant radiotherapy significantly increases the likelihood of major bowel dysfunction [5, 8]. So far, only a few studies investigated the impact of pelvic intraoperative neuromontoring (pIONM) on ano(neo-)rectal functional outcome and reported short-term data [9–11].

The present prospective study investigated the potential impact of related risk factors and pIONM on the incidence of FI within a 2-year follow-up (FU) period.

## Methods

### Participants

Out of a consecutive series of 187 prospectively investigated patients undergoing elective TME for primary rectal cancer between January 2008 and October 2015, 52 were included. Of those 52 patients, 29 underwent pIONM-controlled surgery (within a monocentric clinical trial, “IKONA” ISRCTN06042867—translational research project) [12]. The study excluded patients undergoing abdominoperineal excision, Hartmann’s procedure, and those with T4 rectal cancer, postoperative adjuvant chemoradiotherapy, or missing FU on ano(neo-)rectal function. Patients undergoing pIONM within the prospective randomized controlled multicenter trial “NEUROS” (ClinicalTrials.gov: NCT01585727) were also excluded [13].

In the present study, all patients underwent standardized nerve-sparing TME with dissection in front of Denonvilliers’ fascia carried out by a colorectal surgeon. Those patients with indication for neoadjuvant chemoradiotherapy were treated using 50 Gy in 5 weeks with accompanying chemotherapy followed by surgery after 6 to 8 weeks.

### Pelvic intraoperative neuromonitoring

**Fig. 1:**
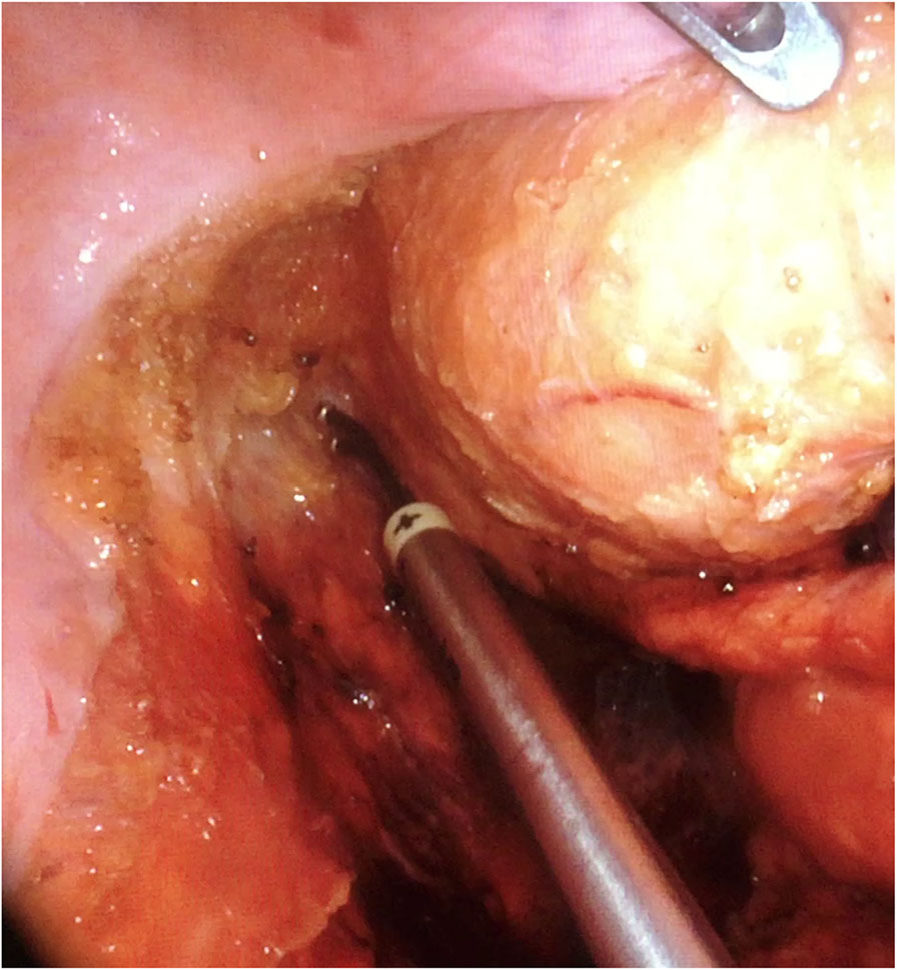
Bipolar electrical stimulation of the pelvic splanchnic nerves during lateral mesorectal dissection

**Fig. 2:**
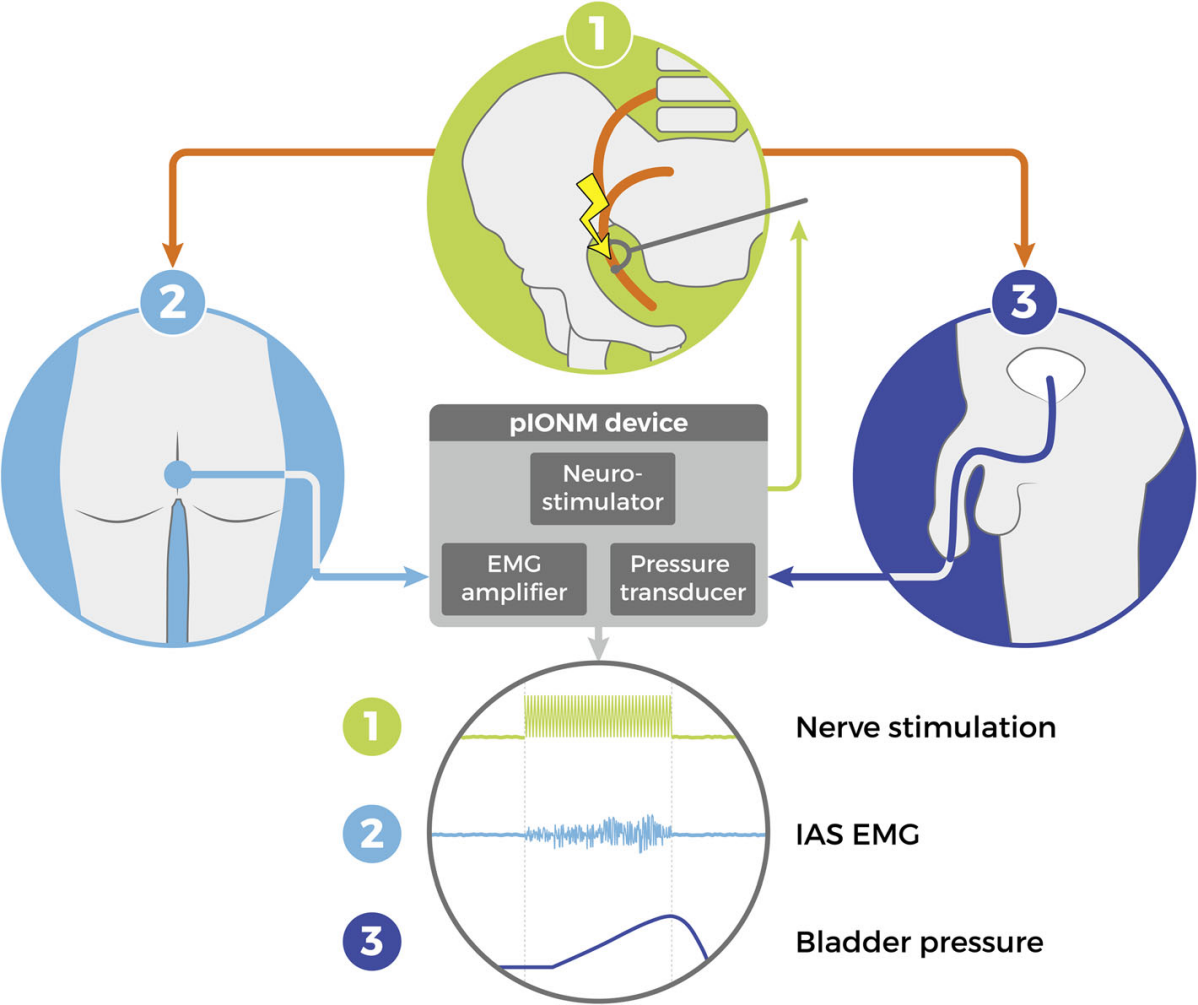
Pelvic intraoperative neuromonitoring (pIONM). EMG electromyography, IAS internal anal sphincter muscle

The pIONM was performed using the standard methodological setup [12]. Medical engineers and an assistant trained in pIONM gave support. The method did not require any additional nerve dissection and was performed during the different steps of TME. Repetitive electric stimulations were carried out to map the autonomic nerves at different sites along the pelvic side and above the level of the pelvic floor. This ensured adequate nerve identification and functional verification during the operation. Stimulations were performed with a hand-guided probe right after posterior dissection to identify the pelvic splanchnic nerves and exposed nerve fibers of the inferior hypogastric plexus. Additional stimulations were performed after lateral/anterolateral dissection and full mobilization of the rectum (Fig. 1). Finally, bilateral repetitive stimulations were carried out after rectal resection for quality control of pelvic autonomic nerve preservation. Currents of 6 mA, frequency of 30 Hz, and monophasic rectangular pulses of 200 μs were used. The stimulations were observed under simultaneous cystomanometry and online-processed electromyography of the internal anal sphincter. Signals were continuously visualized on the monitor of the system (Fig. 2).

### Patient-reported outcome measures

For assessment of the anorectal function, patients were asked to complete the validated Wexner Score. The scoring system ranges from 0 to 20 points and consists of five items, including incontinence of flatus, incontinence of liquid, incontinence of solid, wearing a pad, and lifestyle alteration requirements [14]. Anorectal dysfunction with diminished quality of life was defined by a Wexner Score > 9 [15].

The median time interval between TME and stoma closure was 3 months. Preoperative data was compared to functional outcome at 3 and 6 months after stoma closure (FU1 and FU2). Further, FU were performed at 12 and 24 months after surgery (FU3 and FU4).

### Statistical analysis

The data was analyzed using SPSS 22.0 software (SPSS Inc., Chicago, IL, USA). The influence of predictor variables on the risk of new onset of FI following surgery was calculated using univariate analysis. Functional data was transformed into a binary outcome (new onset of FI vs. no new onset of FI). Variables significantly associated with FI in the univariate analysis were included in a logistic regression analysis in order to examine their independent influence. For comparison of function between the non-pIONM and pIONM group, the chi-square test or Mann-Whitney *U* test was used. Statistical significance was defined as *p* < 0.05.

## Results

Table 1 shows the demographic, clinical, and histopathological details of the patients. None of the patients underwent prior proctological procedures. The endorectal ultrasound revealed neither sphincter defects nor infiltration by the tumor. The baseline Wexner Scores of the non-pIONM and pIONM group were similar (median Wexner Score 0 vs. 0 (*p* = 0.461)).

**Table 1:** Patients’ characteristics

	Non-pIONM group (*n* = 23)	pIONM group (*n* = 29)	*p*
Sex, M/F	12/11	26/3	0.003
Age, years	64 (58, 73)	63 (55, 74)	0.768
Body mass index, kg m^-2^	26 (23, 31)	26 (24, 31)	0.775
ASA classification, I/II/III/IV	2/15/5/1	1/16/12/0	0.308
pT-category (*n*)			0.828
yT0	1	0	
T1 (yT1)	3 (0)	4 (2)	
T2 (yT2)	5 (2)	5 (5)	
T3 (yT3)	10 (2)	5 (8)	
UICC classification (*n*)			0.326
I	9	14	
II	8	4	
III	2	5	
IV	4	6	
Tumor site (*n*)			0.365
Middle rectal third (< 6 cm from anal verge)	17	19	
Lower rectal third (6 to ≤ 12 cm from anal verge)	6	10	
Anterior quadrant involvement (*n*)	19	20	0.211
Neoadjuvant CRT	6	12	0.196
Open/laparoscopic	21/2	24/5	0.318
Stapled anastomosis (*n*)			0439
Colorectal	18	21	
Coloanal	5	8	
Reconstruction (*n*)			0.101
End-to-End	7	12	
Side-to-End	8	14	
J-Pouch	8	3	
Intraoperative blood loss, ml	300 (100, 600)	500 (50, 750)	0.773
Blood transfusion, units	0 (0, 0)	0 (0, 0)	0.768
Anastomotic leakage (*n*) ^a^	0	1	0.558
pR0, pR2 (*n*)	19, 4	23, 6	0.525
pCRM negative, > 1 mm (*n*)	23	29	
M.E.R.C.U.R.Y. Graduation (*n*)			0.588
I°, complete	22	27	
II°, nearly complete	1	2	
Local recurrence	0	0 k	

Values are reported as median (interquartile range) or the number of patients

*M* male, *F* female, *ASA* American Society of Anesthesiologists, *UICC* Union Internationale Contre le Cancer, *LAR* low anterior resection, *pIONM* pelvic intraoperative neuromonitoring, *CRM* circumferential resection margin involvement

^a^Managed conservatively; statistical significance was defined as *p*<0.05

No death occurred within 30 days following surgery. During the further follow-up, two patients died of rectal cancer. Four patients had a history of pelvic surgery (transurethral resection of the prostate (*n* = 2), cystoprostatectomy (*n* = 1), and hysterectomy (*n* = 1)).

Of 52 patients, 10 (19%) reported onset of FI after 3 months following stoma closure (FU1). Six months after stoma closure (FU2), 12 patients (23%) developed FI. One year after surgery (FU3), 16 of 52 (31%) suffered from onset of FI. After 2 years (FU4), 17 of the remaining 50 patients (34%) reported disturbed function.

In the univariate analysis, non-performance of pIONM was associated with an increased risk for onset of FI at short-term FU. At the 1- and 2-year FU, neoadjuvant chemo-radiotherapy, absence of pIONM, and tumor site in the lower rectal third were found to significantly increase the risk for FI (Table 2). In the logistic regression analysis, all identified risk factors remained significant predictors (Table 3).

**Table 2:** Univariate analysis with newly developed fecal incontinence after total mesorectal excision for rectal cancer

Potential	3 months		6 months		12 months		24 months	
Risk factors	After SC	*p*	After SC	*p*	Post-OP	*p*	Post-OP	*p*
Sex								
F	4 of 14	5 of 14	2 of 14	2 of 14				
M	6 of 38	0.254	7 of 38	0.172	14 of 38	0.108	15 of 36	0.063
Age (years)								
≤ 75	8 of 45	9 of 45	12 of 45	13 of 44				
> 75	2 of 7	0.406	3 of 7	0.192	4 of 7	0.120	4 of 6	0.093
Tumor site								
Lower rectal third	3 of 16		5 of 16		9 of 16		9 of 16	
Middle rectal third	7 of 36	0.636	7 of 36	0.277	7 of 36	0.011*	8 of 34	0.026*
Ant. quadrant involvement								
No	2 of 13		3 of 13		4 of 13		4 of 13	
Yes	8 of 39	0.518	9 of 39	0.635	12 of 39	0.627	13 of 37	0.529
Neoadjuvant CRT								
No	8 of 34		9 of 34		7 of 34		8 of 33	
Yes	2 of 18	0.244	3 of 18	0.332	9 of 18	0.032*	9 of 17	0.044*
Approach								
Open	10 of 45		12 of 45		15 of 45		15 of 43	
Laparoscopic	0 of 7	0.202	0 of 7	0.139	1 of 7	0.295	2 of 7	0.554
Intraoperative blood loss (ml)								
≤ 1000	8 of 45		10 of 45		14 of 45		15 of 43	
> 1000	2 of 7	0.406	2 of 7	0.516	2 of 7	0.633	2 of 7	0.554
pIONM								
Yes	2 of 29		3 of 29		5 of 29		6 of 28	
No	8 of 23	0.014*	9 of 23	0.017*	11 of 23	0.019*	11 of 22	0.035*
Mesorectal thickness (cm)^*^								
< 6	7 of 40		7 of 40		11 of 40		12 of 38	
≥ 6	3 of 12	0.418	5 of 12	0.091	5 of 12	0.277	5 of 12	0.378
Tumor size								
≤ 4 cm	7 of 35		8 of 35		11 of 35		12 of 33	
> 4 cm	3 of 17	0.579	4 of 17	0.608	5 of 17	0.574	5 of 17	0.434
pT-category								
(y)pT 0-2	5 of 27		7 of 27		10 of 27		10 of 26	
(y)pT3	5 of 25	0.584	5 of 25	0.431	6 of 25	0.237	7 of 24	0.347
UICC IV								
No	9 of 42		11 of 42		14 of 42		14 of 40	
Yes	1 of 10	0.375	1 of 10	0.261	2 of 10	0.341	3 of 10	0.539
Anastomotic leakage								
No	10 of 51		12 of 51		16 of 51		17 of 49	
Yes	0 of 1	0.808	0 of 1	0.769	0 of 1	0.692	0 of 1	0.660

*SC* stoma closure, *F* female, *M* male, *CRT* chemoradiotherapy, *pIONM* pelvic intraoperative neuromonitoring, *UICC* Union Internationale Contre le Cancer

*Largest cross-section diameter measured by a pathologist on the fixed specimen. Statistical significance was defined as *p* < 0.05

**Table 3:** Independent risk factors for postoperative onset of fecal incontinence assessed by logistic regression analysis

	Relative risk † 3 months After SC	*p*	Relative risk † 6 months After SC	*p*	Relative risk † 12 months Post-OP	*p*	Relative risk † 24 months Post-OP	*p*
Neoadjuvant CRT	–	–	–	–	20.1 (2.7; 166.2)	0.004	9.7 (1.7; 55.3)	0.011
Non-performed pIONM	7.2 (1.4; 38.4)	0.021	5.6 (1.3; 24.0)	0.021	27.3 (3.2; 232.4)	0.002	11.0 (1.9; 63.4)	0.007
Tumor in the lower rectal third	–	–	–	–	25.5 (3.1; 207.4)	0.002	10.8 (1.9; 62.5)	0.008

*CRT* chemoradiotherapy, *pIONM* pelvic intraoperative neuromonitoring

^†^95% confidence intervals; statistical significance was defined as *p*<0.05

**Fig. 3:**
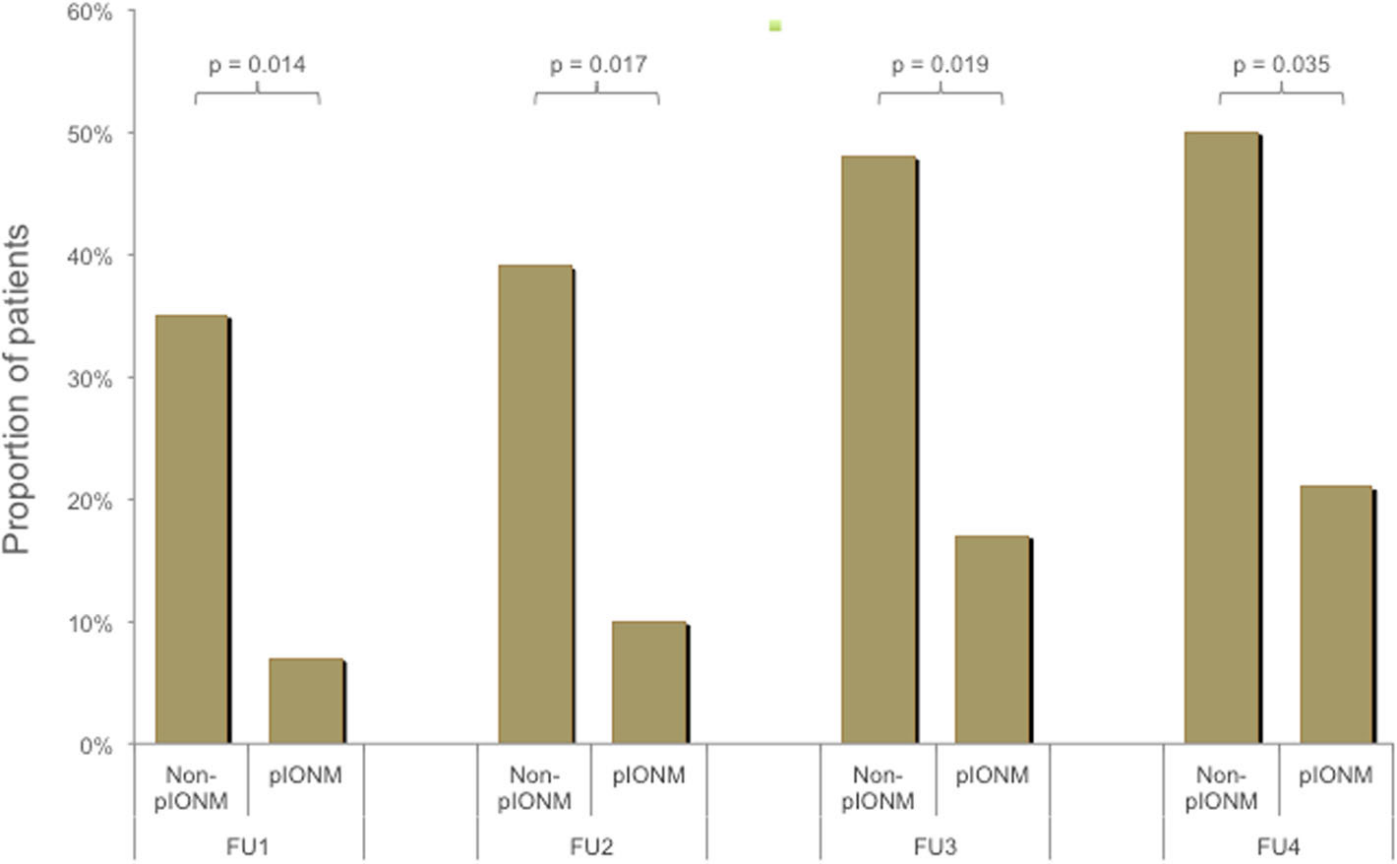
Newly developed fecal incontinence after total mesorectal excision with and without pelvic intraoperative neuromonitoring (pIONM). Ano(neo-)rectal function was prospectively assessed at 3 and 6 months after stoma closure (follow-up (FU) 1 and FU2). Further follow-ups were performed at 12 and 24 months after surgery (FU3 and FU4)

At each FU, the pIONM group had significantly lower rates of newly developed FI than the non-pIONM group (Fig. 3). After the first FU, 2 of 29 patients (7%) had newly developed FI in the pIONM group and 8 of 23 (35%) in the non-pIONM group (*p* = 0.014). At second FU, 3 of 29 (10%) had onset of FI in the pIONM group and 9 of 23 (39%) in the non-pIONM group (*p* = 0.017). After 1 and 2 years, 5 of 29 (17%) and 6 of 28 patients (21%) undergoing pIONM reported onset of FI while in the non-pIONM group 11 of 23 (48%) and 11 of 22 (50%) had developed FI (*p* = 0.019 and *p* = 0.035).

## Discussion

The present study demonstrated that up to one third of the patients undergoing TME for rectal cancer suffer from FI with a diminished quality of life even 2 years after surgery. This is in accordance with the data of a previous meta-analysis on long-term gastrointestinal functional outcomes following curative anterior resection for rectal cancer. This meta-analysis revealed a pooled incidence of FI of approximately 35% ranging from 3.2 to 79.3%. The reported variations might be explained by the use of modified instruments in the vast majority of the analyzed 48 studies despite the availability of validated scoring systems [16].

Emmertsen and Laurberg developed a valid scoring system, the LARS Score, measuring in addition to FI further functional components such as urgency, frequency, and stool clustering in order to determine the true impact of bowel dysfunction [17]. However, at the time of the functional assessment in this study, this scoring system had not been available yet. A recent cross-sectional review of 234 studies between 2004 and 2015 proved the most frequently used instrument to be the Wexner Score, although the LARS Score is gaining popularity [4].

TME under pIONM was found to result in significantly lower rates of FI compared to those undergoing surgery alone (Fig. 3). Similar findings were reported by a previous case-control study presenting short-term data of pIONM-controlled TME compared to TME alone (~ 7% vs. 40%) [9]. Another retrospective study also demonstrated within a short-term FU that pIONM-controlled preservation of the pelvic autonomic nerves maintains fecal continence. Moreover, the authors showed a trend towards higher Wexner Scores when pIONM had not verified nerve integrity [11].

This present study further highlights the effect of pIONM on functional preservation not only being significant in the short course, but also in the subsequent FU, which revealed its sustainability even after 2 years (21% vs. 50%). This finding is in accordance with a recent study analyzing pIONM-controlled TME vs. TME alone for preservation of urinary and sexual function (20% vs. 51% for minor/major urinary dysfunction and 56% vs. 90% for minor/major sexual dysfunction after a 2-year FU) [18].

The positive effect of pIONM on function might result from improved identification of the pelvic autonomic nerves compared to visual assessment alone. A previous study revealed identification rates to be almost twice as high under the use of pIONM (~ 80% vs*.* 45%) [19]. Without identification, the adjacent nervous tissue is at risk to be damaged. This may result in impaired function. However, apparently, the pIONM supports the surgeon’s ability to sense and trace the course of autonomic nerve fibers and thus preserves function. Besides the complexity of this neural network, the identification under visual assessment alone is further limited by a narrow and deep pelvis, voluminous mesorectum, bulky, and low-lying tumors as well as radiotherapy-related scarring [18, 20].

Several studies demonstrated the negative impact of neoadjuvant radiotherapy on ano(neo-)rectal function. Pollak and colleagues revealed FI rates to be more than twice as high in patients undergoing preoperative short-course radiotherapy than in those undergoing surgery alone (57% vs. 26%) [21]. The Dutch trial reported even higher rates in the irradiated group compared to the nonirradiated group (62% vs. 38%) [8]. Another randomized trial compared short-course and long-course radiotherapy and reported no significant difference in the ano(neo-)rectal dysfunction rates, which affected two thirds of the patients in both groups [22]. The reported rates in these randomized trials have to be handled with caution as the instruments used for evaluating ano(neo-)rectal function were not validated, limiting their significance. However, the present study supports the abovementioned previous results. Moreover, this study proved the negative effect of neoadjuvant long-course radiotherapy becoming evident 1 year after surgery, remaining an independent predictor in the long run. Similarly, a previous report demonstrated neoadjuvant long-course radiotherapy to be an independent predictor for onset of urinary and sexual dysfunction 1 and 2 years after TME [18].

In the present study, localization of cancer in the lower rectal third was also found to predict for FI (Table 3), which is in accordance with previous findings [8, 17]. A multicenter study by Battersby and colleagues proved the combination of the predictors “low rectal cancer” and “preoperative radiotherapy” to result in a 60% risk for major bowel-related quality of life impairment compared to 33% for patients with cancer in the middle and upper rectal third and no preoperative radiotherapy [5].

Limitations of the present study are the relatively small sample size, the non-randomized design, and the resulting potential for selection bias. Moreover, the patients’ responses to the questionnaires may have been influenced by the information that surgery was carried out with pIONM. The evaluation of ano(neo-)rectal function was based on the validated Wexner Score, which is suitable for the assessment of the degree of FI, but does not provide information on frequency, urgency, and stool clustering. The LARS Score was not used. In addition, this report is limited to the investigation of the impact of long-course radiotherapy.

## Conclusions

The striking finding of the present study is that performing pIONM reduces the incidence of FI not only in the short course, but also in the further course 2 years after TME. The negative impact of neoadjuvant long course radiotherapy became evident 1 year after surgery and remained an independent predictor in the long run.

Rectal cancer patients need to be informed about potential functional deterioration and side effects of neoadjuvant radiotherapy in order to carefully weigh up the risk of local recurrence and bowel dysfunction. The patients’ expectations on post-treatment quality of life should thus be considered during the decision-making process in the multidisciplinary tumor board. Further investigation could focus on the value of pIONM in patients selected for radiotherapy and how this would affect the functional outcome.

## Data Availability

The datasets used and/or analyzed during the current study are available from the corresponding author on reasonable request.

## Abbreviations

FI: Fecal incontinence
FU: Follow-up
LARS: Low anterior resection syndrome
pIONM: Pelvic intraoperative neuromonitoring
TME: Total mesorectal excision
